# Impact of maternal obesity on neonatal outcomes and birth complications

**DOI:** 10.6026/973206300221700

**Published:** 2026-03-31

**Authors:** Prashant Solanke, Poonam Patil, Galo David Moreno Pineda

**Affiliations:** 1 Department of Community Medicine, Dr. Ulhas Patil Medical College, Jalgaon, Maharashtra, India; 2 Department of Obstetrics and Gynaecology, KIMS Hospital, Minister Road, Secunderabad, India; 3 Department of Medicine, Universidad de las Americas Puebla, San Andrés Cholula, Puebla, Mexico

**Keywords:** Maternal obesity, neonatal outcomes, birth complications, macrosomia, neonatal morbidity

## Abstract

Maternal obesity is a growing global health concern, significantly linked to adverse pregnancy outcomes. Therefore, it is of interest to evaluate the impact of maternal obesity on neonatal outcomes and birth complications compared to non-obese women. Obese mothers had higher rates of cesarean sections, prolonged labor and complications like shoulder dystocia and neonatal morbidities. Neonates of obese mothers were more likely to experience macrosomia, low Apgar scores and NICU admission. Thus, the need for early identification and targeted interventions is needed to improve maternal and neonatal health outcomes.

## Background:

Maternal obesity has emerged as one of the most significant public health challenges of the 21st century, with its prevalence increasing at an alarming rate across both developed and developing countries. Obesity during pregnancy is commonly defined using body mass index (BMI), where a BMI of ≥30 kg/m^2^ prior to conception or during early pregnancy classifies a woman as obese [1]. According to the World Health Organization, the global prevalence of obesity has nearly tripled since 1975 and women of reproductive age represent a substantial proportion of this affected population. This rising trend has profound implications for maternal health, fetal development and neonatal outcomes, making maternal obesity a critical area of concern for clinicians, researchers and policymakers [2]. Pregnancy is a unique physiological state characterized by complex metabolic, hormonal and cardiovascular adaptations that support fetal growth and development. In obese women, these physiological changes are often exaggerated or dysregulated, leading to an increased risk of pregnancy-related complications [3]. Excess adipose tissue is associated with chronic low-grade inflammation, insulin resistance, altered lipid metabolism and endothelial dysfunction. These pathophysiological mechanisms can adversely affect placental function, uteroplacental blood flow and nutrient transport, thereby influencing both maternal and fetal outcomes. As a result, maternal obesity is increasingly recognized not merely as a background characteristic, but as an independent risk factor that significantly modifies the course of pregnancy and childbirth [4]. A growing body of evidence indicates that maternal obesity is strongly associated with a range of adverse obstetric outcomes. Obese pregnant women are at higher risk of developing gestational diabetes mellitus, hypertensive disorders of pregnancy including preeclampsia, thromboembolic events and prolonged or dysfunctional labor [5]. These complications often necessitate medical interventions such as labor induction or operative delivery. Consequently, rates of cesarean section are consistently reported to be higher among obese women compared to those with normal BMI. In addition, obesity increases the likelihood of anesthesia-related difficulties, postpartum hemorrhage, wound infection and delayed recovery, further contributing to maternal morbidity [6]. The impact of maternal obesity extends beyond the mother and has significant consequences for the fetus and the newborn. Neonates born to obese mothers are at increased risk of being large for gestational age (LGA) and macrosomic due to excessive transplacental transfer of glucose, lipids and amino acids.

Fetal overgrowth not only complicates labor and delivery but also predisposes infants to birth injuries such as shoulder dystocia, brachial plexus injury and clavicular fractures [7]. Conversely, maternal obesity has also been associated with fetal growth restriction in certain contexts, possibly due to placental insufficiency, highlighting the complex and sometimes paradoxical effects of obesity on fetal growth patterns [8]. Neonatal outcomes related to maternal obesity are of particular concern, as they have both immediate and long-term health implications. Infants born to obese mothers are more likely to experience low Apgar scores, respiratory distress, meconium aspiration, hypoglycemia and admission to neonatal intensive care units (NICUs). These early-life complications contribute to increased neonatal morbidity, prolonged hospital stays and higher healthcare costs [9]. Moreover, emerging evidence suggests that the intrauterine environment associated with maternal obesity may induce epigenetic modifications that program the offspring for future metabolic disorders, including childhood obesity, type 2 diabetes mellitus and cardiovascular disease. This concept of developmental origins of health and disease underscores the intergenerational impact of maternal obesity [10]. Birth complications related to maternal obesity pose additional challenges in obstetric practice. Excess maternal weight can impair accurate clinical assessment, including fetal monitoring and ultrasound evaluation, leading to diagnostic and management difficulties. Prolonged labor, failed induction and increased operative delivery rates are frequently reported in obese women, increasing the risk of maternal and neonatal trauma [11]. Shoulder dystocia, in particular, is a feared complication strongly linked to fetal macrosomia and maternal obesity, requiring skilled and timely intervention to prevent serious neonatal injury or death. These complications highlight the need for careful intrapartum management and risk stratification in obese pregnant women [12]. Despite growing awareness, significant gaps remain in understanding the full extent of the impact of maternal obesity on neonatal outcomes and birth complications, particularly in low- and middle-income countries where the dual burden of under-nutrition and obesity coexists [13]. Variations in study design, population characteristics and definitions of obesity and outcome measures have resulted in inconsistent findings across the literature. Additionally, confounding factors such as maternal age, parity, socioeconomic status, gestational weight gain and access to antenatal care further complicate the interpretation of existing evidence. Addressing these gaps is essential for developing evidence-based guidelines and targeted interventions aimed at improving maternal and neonatal health outcomes [14]. In this context, there is a pressing need for well-designed studies that comprehensively evaluate the relationship between maternal obesity, neonatal outcomes and birth complications within specific populations. Understanding these associations will aid in early risk identification, optimization of antenatal care and formulation of preventive strategies, including preconception counseling and appropriate weight management programs during pregnancy. Furthermore, generating population-specific evidence is essential for guiding clinical decision-making and informing public health policies aimed at reducing obesity-related perinatal risks [15]. Therefore, it is of interest to assess the impact of maternal obesity on neonatal outcomes and birth complications in order to facilitate timely interventions and improve overall perinatal care.

## Methodology:

## Study design and setting:

This original research was designed as a hospital-based observational analytical study conducted in the Department of Obstetrics and Gynecology of a tertiary care teaching hospital. The study was carried out over a defined period of 12 months to evaluate the impact of maternal obesity on neonatal outcomes and birth complications.

## Study population and sample size:

A total sample size of 100 pregnant women was included in the study. The sample size was selected based on feasibility, availability of eligible participants during the study period and consistency with similar previously published studies assessing maternal BMI and perinatal outcomes. Participants were recruited consecutively until the required sample size was achieved.

## Inclusion criteria:

[1] Pregnant women aged 18-40 years

[2] Singleton pregnancy

[3] Gestational age ≥37 weeks at the time of delivery

[4] Women with documented pre-pregnancy or first-trimester body mass index (BMI)

[5] Willingness to participate and provide informed consent

## Exclusion criteria:

[1] Multiple gestations

[2] Pregnancies complicated by major fetal congenital anomalies

[3] Women with pre-existing chronic medical disorders such as pregestational diabetes mellitus, chronic hypertension, renal disease, or cardiac disease

[4] Women with incomplete medical records

## Grouping of participants:

Based on BMI calculated at the first antenatal visit, participants were categorized into two groups:

[1] Obese group (n = 50): BMI ≥30 kg/m^2^

[2] Non-obese group (n = 50): BMI 18.5-24.9 kg/m^2^

BMI was calculated using the formula: weight (kg) / height (m^2^). Standard BMI classification criteria were used for grouping.

## Data collection:

Data were collected using a structured proforma through patient interviews, clinical examination and review of medical records. Maternal variables recorded included age, parity, BMI, gestational age at delivery, mode of onset of labor, mode of delivery and intrapartum complications such as prolonged labor, shoulder dystocia, postpartum hemorrhage and need for operative intervention. Neonatal outcomes assessed included birth weight, Apgar scores at 1 and 5 minutes, presence of macrosomia or low birth weight, need for neonatal resuscitation, respiratory distress, hypoglycemia, meconium aspiration and admission to the neonatal intensive care unit (NICU).

## Outcome measures:

[1] Primary outcomes: Neonatal birth weight, Apgar scores, NICU admission

[2] Secondary outcomes: Mode of delivery, birth complications (*e.g.*, shoulder dystocia) and immediate neonatal morbidity

## Statistical analysis:

Data were entered into Microsoft Excel and analyzed using statistical software. Continuous variables were expressed as mean ± standard deviation, while categorical variables were expressed as frequencies and percentages. The independent t-test was used to compare continuous variables between groups and the chi-square test or Fisher's exact test was used for categorical variables. A p-value of <0.05 was considered statistically significant.

## Ethical considerations:

Ethical approval was obtained from the Institutional Ethics Committee prior to commencement of the study. Written informed consent was obtained from all participants. Confidentiality of patient information was strictly maintained and the study was conducted in accordance with ethical principles for medical research involving human subjects.

## Results:

A total of 100 pregnant women were included in the study, of which 50 were classified as obese (BMI ≥30 kg/m^2^) and 50 as non-obese (BMI 18.5-24.9 kg/m^2^). Maternal characteristics, birth complications and neonatal outcomes were compared between the two groups.

Maternal obesity was significantly associated with higher maternal age, increased rates of cesarean section, prolonged labor and shoulder dystocia (Tables 1 and 2 - see PDF). Neonates born to obese mothers had a significantly higher incidence of macrosomia (Table 3 - see PDF), lower Apgar scores and increased need for resuscitation (Table 4 - see PDF). Furthermore, neonatal morbidity-including respiratory distress, hypoglycemia and NICU admission-was significantly more common among infants of obese mothers (Table 5 - see PDF). These findings indicate a strong association between maternal obesity and adverse neonatal outcomes as well as increased birth complications.

## Discussion:

The findings of this study demonstrate a significant association between maternal obesity and adverse neonatal outcomes and birth complications, aligning closely with a robust body of evidence from previously published research. In our study, obese mothers had significantly higher rates of cesarean delivery, prolonged labor and shoulder dystocia compared with non-obese women. Neonates born to obese mothers were more likely to be macrosomic, have lower Apgar scores, require resuscitation at birth and experience neonatal complications such as respiratory distress, hypoglycemia and NICU admission. These results are consistent with the systematic review by Weir *et al.* (2024) [16], which showed that maternal obesity is strongly associated with increased risks of macrosomia, large-for-gestational-age infants and shoulder dystocia. Their pooled analysis highlighted that excess maternal weight alters fetal growth patterns and labor dynamics, leading to both obstetric and neonatal challenges similar to those observed in our cohort. Similarly, Lutsiv *et al.* (2015) [17] conducted a meta-analysis of multiple cohort studies and reported that morbid obesity significantly increased risks of preterm birth, large-for-gestational-age infants and several adverse neonatal outcomes compared to women with normal BMI. This supports our findings of higher frequencies of macrosomia and subsequent neonatal morbidity in the obese group, indicating that the severity of obesity may have a pronounced effect on both fetal growth and early neonatal health. In an observational study, Avci *et al.* (2015) [18] found that maternal obesity was associated with increased rates of low Apgar scores, NICU admissions and neonatal hypoglycemia, outcomes that closely mirror our results. Their study highlighted that metabolic dysregulation in obese pregnancies likely contributes to immediate postnatal adaptation challenges, which was also evident in the significantly higher incidence of low Apgar scores and need for resuscitation in neonates born to obese mothers in our cohort. The cohort analysis by Roman *et al.* (2011) [19] specifically examined women with gestational diabetes and obesity, reporting an increased incidence of stillbirth, shoulder dystocia, macrosomia, neonatal hypoglycemia and NICU admission among obese mothers. Although our study did not exclusively include diabetic pregnancies, the similarity in observed neonatal risks reinforces the fact that maternal obesity independently contributes to adverse outcomes, possibly exacerbated further by underlying metabolic conditions. More recently, Dinsmoor *et al.* (2023) [20] conducted a large multicenter cohort study with a focus on morbid obesity and found significantly elevated odds of composite neonatal morbidity, including respiratory difficulty, hypoglycemia and NICU care, compared with reference BMI groups. These findings are comparable to our results, where obese mothers had significantly higher rates of NICU admission and neonatal complications, supporting the conclusion that as maternal BMI increases, so does the likelihood of adverse neonatal outcomes. Collectively, these studies, along with our own data, underscore that maternal obesity is a major risk factor for a range of unfavorable birth outcomes and early neonatal complications. The consistency of these findings across varied populations and study designs strengthens the evidence base recommending enhanced prenatal care for obese women. Clinicians should focus on early BMI assessment, targeted nutritional counseling and surveillance strategies aimed at minimizing risks such as macrosomia and metabolic disturbances. Furthermore, these data suggest that interventions to optimize maternal weight before and during pregnancy could significantly improve perinatal outcomes. Given the potential for maternal obesity to contribute to both short-term neonatal morbidity and long-term health risks, greater emphasis should be placed on preconception health programs, public health messaging and policy initiatives that address obesity among women of reproductive age. Future research with larger, diverse samples and standardized outcome measures will be important to further refine risk stratification and intervention strategies for this high-risk population.

## Conclusion:

Maternal obesity is strongly linked to increased birth complications and adverse neonatal outcomes, including higher rates of cesarean delivery, prolonged labor and shoulder dystocia. Neonates of obese mothers are more likely to experience macrosomia, low Apgar scores and metabolic complications. Early identification and targeted antenatal interventions are essential to improve perinatal outcomes.
